# Invasive Aquatic Plants as Ecosystem Engineers in an Oligo-Mesotrophic Shallow Lake

**DOI:** 10.3389/fpls.2018.01781

**Published:** 2018-12-03

**Authors:** Cristina Ribaudo, Juliette Tison-Rosebery, Damien Buquet, Gwilherm Jan, Aurélien Jamoneau, Gwenaël Abril, Pierre Anschutz, Vincent Bertrin

**Affiliations:** ^1^EA 4592 Géoressources et Environnement, ENSEGID, Pessac, France; ^2^Irstea, UR EABX, Centre de Bordeaux, Cestas, France; ^3^CNRS UMR 5805 Environnements et Paléoenvironnements Océaniques et Continentaux, Université de Bordeaux, Pessac, France; ^4^Biologie des Organismes et Ecosystèmes Aquatiques, Muséum National d’Histoire Naturelle, Paris, France; ^5^Programa de Geoquímica, Universidade Federal Fluminense, Niterói, Brazil

**Keywords:** carbon emission, methane, hypoxia, water stratification, nutrients regeneration, seasonal, primary production, exotic plants

## Abstract

Exotic hydrophytes are often considered as aquatic weeds, especially when forming dense mats on an originally poorly colonized environment. While management efforts and research are focused on the control and on the impacts of aquatic weeds on biodiversity, their influence on shallow lakes’ biogeochemical cycles is still unwell explored. The aim of the present study is to understand whether invasive aquatic plants may affect the biogeochemistry of shallow lakes and act as ecosystem engineers. We performed a multi-year investigation (2013–2015) of dissolved biogeochemical parameters in an oligo-mesotrophic shallow lake of south-west of France (Lacanau Lake), where wind-sheltered bays are colonized by dense mats of exotic *Egeria densa* Planch. and *Lagarosiphon major* (Ridl.) Moss. We collected seasonal samples at densely vegetated and plant-free areas, in order to extrapolate and quantify the role of the presence of invasive plants on the biogeochemistry, at the macrophyte stand scale and at the lake scale. Results revealed that elevated plant biomass triggers oxygen (O_2_), dissolved inorganic carbon (DIC) and nitrogen (DIN) stratification, with hypoxia events frequently occurring at the bottom of the water column. Within plants bed, elevated respiration rates generated important amounts of carbon dioxide (CO_2_), methane (CH_4_) and ammonium (NH_4_^+^). The balance between benthic nutrients regeneration and fixation into biomass results strictly connected to the seasonal lifecycle of the plants. Indeed, during summer, DIC and DIN regenerated from the sediment are quickly fixed into plant biomass and sustain elevated growth rates. On the opposite, in spring and autumn, bacterial and plant respiration overcome nutrients fixation, resulting in an excess of nutrients in the water and in the increase of carbon emission toward the atmosphere. Our study suggests that aquatic weeds may perform as ecosystem engineers, by negatively affecting local oxygenation and by stimulating nutrients regeneration.

## Introduction

Global changes, such as the increase of water temperature, the modifications of lakeshore for anthropic activities and the unintentional introduction of plant fragments may favor the spread of exotic aquatic plants (Gillard et al., 2017; Bertrin et al., 2018). The settlement of invasive species, and the massive developed biomass, has been recently recognized to strongly influence biodiversity (Hussner et al., 2009; Strayer, 2010). However, the effect of invasive species on ecosystem functioning is little known and could be either neutral or positive, by triggering significant changes on the basic processes of the ecosystem (Crooks, 2002; Havel et al., 2015).

The presence of invasive macrophytes can strongly modify aquatic local conditions, and particularly the water temperature, the sediment chemistry and the nutrients cycling of the colonized area, especially in the case of rootless or floating-leaved hydrophytes (Urban et al., 2006; Pierobon et al., 2010; Andersen et al., 2017; Vilas et al., 2017). Indeed, the massive plant coverage at the air–water interface is recognized to generate thermal and chemical stratification, even within very shallow waters (Andersen et al., 2017). While submerged rooted macrophytes at moderate biomass are known to increase sedimentary redox potential thanks to radial oxygen loss (Racchetti et al., 2010; Ribaudo et al., 2011), extremely dense submerged canopies may lead to decreased redox potentials and increased benthic nutrients fluxes, as a result of limited water mixing (Boros et al., 2011). As in the case of floating hydrophytes, the oxygen consumption from mineralization of plant detritus may favor the production of anaerobic end-products and nutrients regeneration such as methane and ammonium (Bianchini et al., 2008; Pierobon et al., 2010; Oliveira-Junior et al., 2018).

Benthic nutrients release from densely vegetated sediments is favored by particles trapping by submerged shoots and sediment accretion, and may therefore constitute a functional advantage for plant development (Madsen et al., 2001). Macrophytes will use nutrients regenerated from the sediment for their growth and deplete them, especially in nutrients-poor contexts (Bini et al., 2010). In oligotrophic systems, characterized by a low productivity, the settlement of fast growing primary producers can thus accelerate nutrients cycling and boost organic matter degradation, especially in summer, in correspondence with the maximum growth rates and elevated temperatures. On the opposite, during the senescence of the plants in autumn, respiration processes will be prevailing over nutrients fixation and regenerate nutrients toward the water column (Bowes et al., 1979; Pierobon et al., 2010; Ribaudo et al., 2011, 2012).

The balance between nutrients regeneration from sediments and uptake by plants is a key concept for investigating the net effect of the presence of large macrophytes stands within nutrient-poor shallow lake (Bowes et al., 1979). Indeed, at the lake scale, vegetated littoral zones are recognized as hotspots of primary production that take advantage from watershed nutrients incomes, while nutrients and organic matter outputs to the pelagic zone depend on water currents and wind velocity (Wetzel, 1992). Abundant plant biomass can self-sustain thanks to organic matter accretion and nutrients regeneration even when the input from the watershed is low (Marion and Paillisson, 2003).

In this study, several sampling campaigns were carried out in aquatic weed dense meadows of a shallow oligo-mesotrophic lake, with the aim of understanding whether two invasive aquatic plants can act as ecosystem engineers in a nutrient-poor system (Crooks, 2002). More precisely, we hypothesized that dense invasive macrophytes stands will (i) induce thermal and nutrients stratification in the water column, and (ii) contribute to the regeneration of nutrients from the sediments according to a lifecycle seasonal pattern. To test those hypotheses, we worked at two different spatial scales: (1) at the vegetated stand scale for understanding the role of the two invasive hydrophytes in enhancing nutrients regeneration and (2) at the lake scale, to contextualize the role of massive stands in shallow lakes concerning nutrients and carbon budgets.

## Materials and Methods

### Study Area

Lacanau Lake is one of the oligo-mesotrophic shallow lakes of the French Atlantic Lakes chain, located between the Gironde and the Adour estuaries in South-West of France, together with Carcans-Hourtin, Cazaux-Sanguinet, and Parentis-Biscarrosse lakes (Cellamare et al., 2012; Moreira et al., 2015). French Atlantic Lakes are *Lobelia* shallow lakes, known for being colonized by a few macrophyte species, which are typically distributed along the first meter of the water column. Macrophyte community is here mainly composed by isoetids (*Lobelia dortmanna* L., *Littorella uniflora* (L.) Asch. and *Isoetes boryana* Durieu) together with some species of charophytes (Bertrin et al., 2018). This community is recognized to reduce carbon benthic fluxes and to contribute to sediment oxygenation through radial oxygen loss. Their slow metabolism and low growth rates do not affect biochemical cycles on the short term nor water stratification (Ribaudo et al., 2017). Within those lakes, the nutrients budget is mainly driven by benthic fluxes and input from the small watershed and rainfalls (Buquet et al., 2017).

In French Atlantic Lakes, the strong wind and the oligotrophic conditions do not allow the settlement of large canopy-forming hydrophytes, which typically require still waters, nutrients availability and organic-rich sediments. Nevertheless, since about 40 years, large submerged stands of two caulescent aquatic plants [*Egeria densa* Planch and *Lagarosiphon major* (Ridl.) Moss] have been found in some areas of those lakes. *E. densa* and *L. major* are two non-native hydrophytes belonging to the Hydrocharitaceae family, characterized by long erected stems with alternate or opposed whorled leaves. They preferentially settle and develop in sheltered creeks and ports of the lake, between -0.5 and -3.5 m; sparse shoots could be present until 6 m deep. Within French Atlantic Lakes, those hydrophytes develop a total biomass up to 4000 g_DW_ m^-2^ (Bertrin et al., 2017). They present elevated growth rates, giving them a selective advantage over other hydrophytes species, notably thanks to the presence of adventitious roots allowing vegetative multiplication and large dispersion capacities (Haramoto and Ikusima, 1988). According to available past reports (François, 1948), the two species have not replaced other hydrophytes in French Atlantic Lakes. Indeed, *Myriophyllum* spp. were present in 1940’s with sparse shoots, but never developed such biomass and meadow extent.

Lacanau Lake’s surface is 16.2 km^2^ and the mean depth is 2.6 m, Secchi disk is 3.5 m (Moreira et al., 2015). Within the lake, *E. densa* and *L. major* form dense stands, with total biomass >50 g_DW_ m^-2^ occupying 1.19 km^2^ (about 7% of the lake surface, according to Bertrin et al., 2017). The substrate on which plants develop is composed of a sandy substrate covered by a thick layer of labile organic matter-rich sediment presenting 35 ± 21% (*n* = 59) as loss of ignition (Bertrin et al., 2017).

### Seasonal Vertical Stratification

**FIGURE 1:**
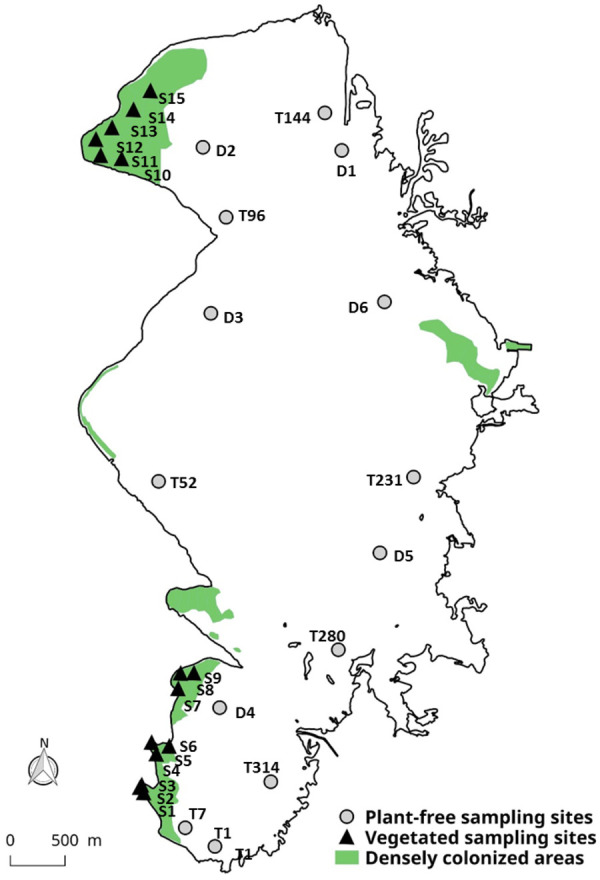
Location of the sampling stations within Lacanau Lake; the correspondence to the sampling sites is reported in Appendix 1. Distribution map of *E. densa* and *L. major* densely colonized areas (>50 g_DW_ m^-2^) refers to 2014 and is taken from Bertrin et al. (2017).

In dense vegetated stands, water sampling was performed at 15 sites between June 2013 and November 2015 (Figure 1 and Appendix 1). For each site, measurements were carried out in duplicates, twice during the day (in the morning, at around 11 a.m. and in the early afternoon, before 3 p.m.) and repeated during the growing seasons in this temperate region of France: spring (March to June), summer (June to September), and autumn (September to November). Water samplings were performed from a boat using a silicone pipe connected to a peristaltic pump. One pipe’s extremity was inserted into the plants stand deep to about 40 cm above the sediment; the other end was connected to a syringe on the boat. Samples within the plants (*Vegetated – Bottom* samples) were collected at depths ranging from 100 to 330 cm, with an average of 249 ± 72 cm. Samples taken just below the water column surface (*Vegetated – Surface* samples) were collected directly from the boat without the use of the pump. During each sampling cycle, the vegetated stand height was systematically measured using a graduated pole.

In unvegetated areas, water sampling was carried out during the day (*n* = 1–6, collected between 10 a.m. and 5 p.m.) between May 2013 and November 2015, at 14 sites where aquatic weeds were totally absent, within parallel studies (Buquet et al., 2017; Ribaudo et al., 2017; Jamoneau, unpublished; Figure 1 and Appendix 1). Samples were collected at the top of the water column (hereafter *Plant-free – Surface* samples) and, where the depth allowed it, samples were also collected at -3 m by using a 2L Niskin bottle, hereafter referred as *Plant-free – Bottom* samples (Buquet et al., 2017).

For each sampling, an aliquot was immediately transferred to a beaker, in which water temperature and pH were measured with a YSI Multiple Probe (model 556). Separated aliquots were sampled in borosilicate vials and then analyzed in the laboratory for dissolved oxygen (O_2_ – by Winkler method), alkalinity (TAlk – by titration with HCl 0.1 M), carbon dioxide [CO_2_ – by retrocalculation starting from TAlk and *in situ* pH, validated by measures of dissolved inorganic carbon, DIC, to verify that organic alkalinity was negligible (Abril et al., 2015)] and methane (CH_4_ – by headspace GC-FID method) analyses, following the methods reported in Ribaudo et al. (2017). A separate aliquot was filtered (Whatman GF/F filters) and transferred to a 50 ml plastic vial for subsequent dissolved inorganic nitrogen (NO_3_^-^ and NH_4_^+^) analyses by ionic chromatography (METROHM 881 – compact). An aliquot of 50 ml was filtered by GF/F filters and transferred to a borosilicate glass flask for measurement of dissolved organic carbon (DOC) by COTmeter. An aliquot of 500 ml was transferred to a PE-HD flask for total phosphorus (TP) measurement by spectrophotometric assay after NF acid mineralization T90-023, and total nitrogen (TN) after selenium mineralization NF EN 25663. TP and TN were measured only at vegetated sites and at the surface of some of plant-free sites (see Appendix 1).

For vegetated stands, we calculated excess dissolved inorganic carbon (eDIC, μM), as the difference between the *in situ* DIC and a theoretical DIC at atmospheric equilibrium (for CO_2_ = 400 ppmv), according to Abril et al. (2000). The apparent oxygen utilization (AOU, μM) was calculated for vegetated stands according to Dinauer and Mucci (2017), as the deviation of oxygen from an O_2_ concentration in equilibrium with the atmosphere.

### Seasonal Nutrients and Carbon Budget

Together with seasonal samplings, plant harvesting was carried out for biomass measurements. An additional winter sample was taken in February 2014 (*n* = 7), in order to obtain a winter value for growth rate calculations. Samples were always collected by the same operator to minimize the sources of error (Johnson and Newman, 2011), using a telescopic rake (ground sampling area = 0.28 m^2^). The plants were kept cold, transported fresh to the laboratory in opaque bags and transferred to water-filled containers until the moment of measurement, in order to facilitate their handling. In laboratory, the dry weight (g_DW_) was determined after 72 h at 70°C and expressed as total biomass (below + aboveground biomass, g_DW_ m^-2^). Number of shoots was counted for obtaining a shoot density (shoots m^-2^).

The gross growth rate (GGR, expressed as g_DW_ g_DW_^-1^d^-1^) was calculated as follows:

$$\begin{array}{c} \mathrm{GGR}=\mathrm{NGR}+\mathrm{abs}(\mathrm{DR}) \end{array}$$

where NGR is the net growth rate (g_DW_ g_DW_^-1^d^-1^), measured at different temperatures for *Egeria* spp. by Haramoto and Ikusima (1988) and Tavechio and Thomaz (2003) corresponding to a value of 0.020, 0.050, 0.030, and 0.005 g_DW_ g_DW_^-1^d^-1^, for spring, summer, autumn, and winter, respectively. DR is the biomass decay rate (g_DW_ g_DW_^-1^d^-1^), measured at different seasons for *Egeria* spp. by Carvalho et al. (2005), Carrillo et al. (2006), and Suzuki et al. (2015), corresponding to a value of 0.016, 0.045, 0.036, and 0.014 g_DW_ g_DW_^-1^d^-1^, for spring, summer, autumn, and winter, respectively.

The GGR, NGR, and DR obtained for each sampling site and season were multiplied by the biomass measured in the same site and season, in order to obtain a daily gross primary production (GPP, g_DW_ m^-2^d^-1^), a daily net primary production (NPP, g_DW_ m^-2^d^-1^) and a daily decomposition (DD, g_DW_ m^-2^d^-1^). Each seasonal value (averaged from *n* = 15 sites) was then multiplied by 90 days (corresponding to one season of 3 months) and then summed in order to obtain an annual estimation. Further, weight production/decomposition was converted into nutrients uptake/loss rates, by using an average content of 0.360 g C g_DW_^-1^, 0.015 g N g_DW_^-1^ and 0.003 g P g_DW_^-1^ in the plant tissue (Carvalho et al., 2005; Yarrow et al., 2009; Suzuki et al., 2015). In this way, we obtained an estimation of the amount of carbon and nutrients fixed/loss annually into/from biomass at the local and at the lake scale by considering a total surface covered by plants of 1.19 km^2^.

Starting from the surface gas concentrations and local wind speed, CO_2_ and CH_4_ diffusive fluxes at the water–air interface were calculated, following the two-layer model proposed by Liss and Slater (1974). Diffusive fluxes at the water–air interface (*F*) were calculated as follows:

$$\begin{array}{c} F=k\times (C_{\mathrm{meas}}-C_{\mathrm{eq}}) \end{array}$$

where *C*_meas_ is the gas concentration measured in the surface sample expressed as mg C L^-1^, *C*_eq_ is the gas concentration in surface sample in equilibrium with the atmosphere (calculated in function of the temperature from Henry’s law – Sander, 1999) and *k* is the gas transfer velocity constant (cm h^-1^). Gas transfer velocity varies in function of turbulence at the water–air interface, which is mostly generated by winds in lakes (Repo et al., 2007; Cole et al., 2010). Gas transfer velocity was calculated using the average wind speed of 3.5 m s^-1^ measured over the lake at all seasons (Bertrin et al., 2017) and the equation of Crusius and Wanninkhof (2003). Fluxes were calculated for CO_2_ and CH_4_ and then summed for obtaining a total carbon flux: *F* was then expressed as mg C m^-2^h^-1^. Calculated fluxes were thus diffusive and did not account for ebullition processes; in our study, CO_2_ and CH_4_ diffusive fluxes merely served to compare the dynamics of C with and without plants. Hourly fluxes were averaged and upscaled for each sampling season (90 days each), and then summed to obtain a budget for the growing season of the plants (March to November). Finally, fluxes calculated for vegetated stands were upscaled to the lake’s surface covered by invasive plants (1.19 km^2^), while fluxes calculated in plant-free areas were upscaled to the unvegetated lake’s surface (15.01 km^2^).

### Statistical Analyses

We tested the influence of the presence/absence of dense vegetated stands on the biogeochemistry of the water column by a three-way ANOVA. The presence/absence of vegetated stands (*Plant presence*, two levels: vegetated vs. plant-free areas), season (*Season*, three levels: spring vs. summer vs. autumn) and sampling depth (*Depth*, two levels: surface vs. bottom) were considered as fixed factors, while sampling site (*Site*, 29 levels) was considered as a random factor. When checking for analysis of variance assumptions, we found that almost every physicochemical parameter was not normally distributed (Shapiro–Wilk test for normality assumption). Nevertheless, considered the width of the dataset and that homogeneity of variances was always attained (Levene’s test for homoscedasticity assumption), we decided not to apply any data transformation. *Post hoc* analyses were performed by Tukey’s Honestly Significant Difference (HSD) test.

In order to test the correlation between physicochemical parameters, linear regressions and Pearson correlation coefficients were performed. Statistical analyses were performed with R Program (R – Development Core Team 2018). Mean values are reported with their standard deviation.

## Results

### Seasonal Vertical Stratification

**FIGURE 2:**
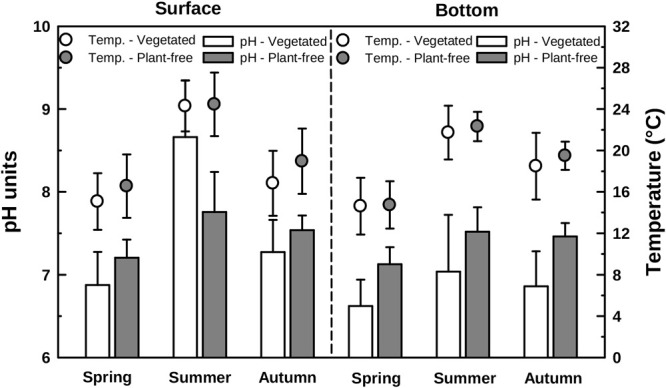
Water pH (bars, left scale) and temperature (points, right scale) variations measured along different seasons in vegetated stands and plant-free areas at the surface and at the bottom of the water column. Error bars represent standard deviation.

Water temperature measured in vegetated stands was significantly lower than that measured in plant-free areas irrespective of the season (annual mean 18.7 ± 4.4 and 20.0 ± 4.3°C, for vegetated and plant-free areas, respectively). At both vegetated and plant-free areas, water temperature varied seasonally (summer higher than spring and autumn; HSD test, *p* < 0.001) (Figure 2 and Table 1). The vegetated stands were thermally stratified in summer (surface warmer than bottom; HSD test, *p* < 0.001), contrary to plant-free sites, which were never stratified. pH differences between vegetated and plant-free sites depended upon the season and the depth (Figure 2). At plant-free areas, differences between surface and bottom were significant only during summer (HSD test, *p* < 0.001), unlike vegetated stands, where the water column was stratified all year around.

**Table 1:** Summarized results of the three-way ANOVA on physicochemical parameters (plant presence, season, and sampling depth as fixed factors; sampling site as random factor). For TN and TP only, a two-way ANOVA was performed (season and sampling depth as fixed factors; sampling site as random factor).

	pH		Temperature		O_2_		CO_2_		CH_4_	
Source	*df*, residuals	*p*	*df*, residuals	*p*	*df*, residuals	*p*	*df*, residuals	*p*	*df*, residuals	*p*
Plant presence	1, 31	0.0596	1, 31	0.0018	1, 31	<0.001	1, 28	<0.001	1, 26	0.0847
Sampling depth	1, 305	<0.001	1, 305	0.6804	1, 478	<0.001	1, 437	<0.001	1, 471	<0.001
Season	1, 305	<0.001	1, 305	<0.001	1, 478	<0.001	1, 437	<0.001	1, 471	0.0550
Plant × Depth	1, 305	<0.001	1, 305	0.4911	1, 478	<0.001	1, 437	<0.001	1, 471	0.0130
Season × Depth	1, 305	<0.001	1, 305	<0.001	1, 478	0.1675	1, 437	0.9790	1, 471	0.3187
Plant × Season	1, 305	<0.001	1, 305	0.1870	1, 478	<0.001	1, 437	0.0312	1, 471	0.6093
Plant × Season × Depth	1, 305	<0.001	1, 305	0.2377	1, 478	0.9595	1, 437	0.9122	1, 471	0.7920

	**NH_4_^+^**		**NO_3_^-^**		**DOC**		**TN**		**TP**	
	***df*, residuals**	***p***	***df*, residuals**	***p***	***df*, residuals**	***p***	***df*, residuals**	***p***	***df*, residuals**	***p***

Plant presence	1, 31	0.4659	1, 31	0.0082	1, 26	<0.001	1,12	–	1, 12	–
Sampling depth	1, 538	<0.001	1, 519	0.0052	1, 216	0.0667	1, 56	0.0497	1, 56	0.4002
Season	1, 538	<0.001	1, 519	<0.001	1, 216	0.0045	1, 56	<0.001	1, 56	0.0141
Plant × Depth	1, 538	0.0046	1, 519	0.9777	1, 216	0.1326	1, 56	–	1, 56	–
Season × Depth	1, 538	0.0287	1, 519	<0.001	1, 216	0.9738	1, 56	0.4862	1, 56	0.6519
Plant × Season	1, 538	0.0220	1, 519	<0.001	1, 216	<0.001	1, 56	–	1, 56	–
Plant × Season × Depth	1, 538	0.0871	1, 519	0.1509	1, 216	0.8363	1, 56	–	1, 56	–

**FIGURE 3:**
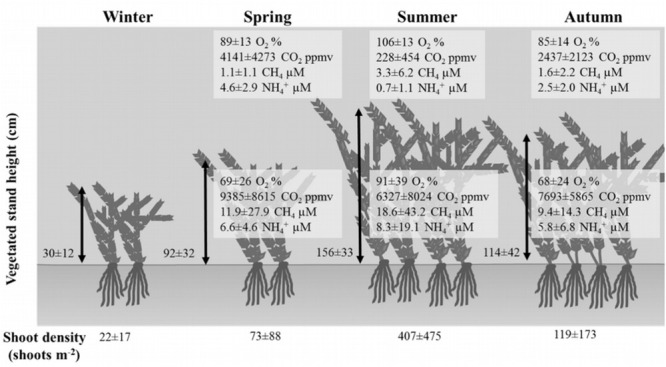
Seasonal variation of some physicochemical parameters in function of plant density and space occupation by invasive aquatic plants. Mean ± SD are reported.

**FIGURE 4:**
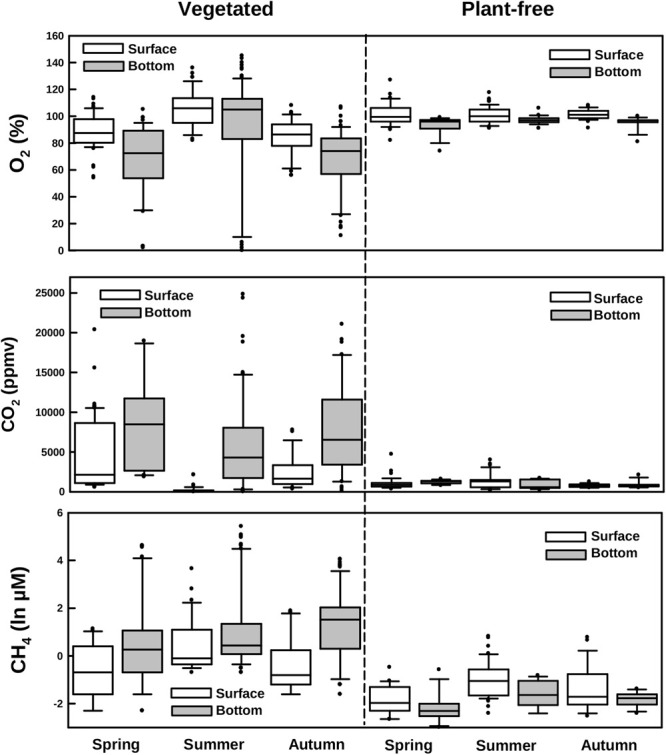
Boxplots of dissolved oxygen, carbon dioxide, and methane measured along different seasons in vegetated and plant-free areas at the surface and the bottom of the water column.

Dissolved oxygen and carbon dioxide significantly varied according to the sampling depth and the season at vegetated sites (Table 1; HSD test, *p* < 0.001), whereas in plant-free areas values were constant along the year and homogenous in the water column (Figures 3, 4; HSD test, *p* < 0.001). At plant-free sites, O_2_ averaged 97 ± 17% and CO_2_ averaged 945 ± 65 ppmv. At vegetated sites, CO_2_ was generally much above 400 ppmv and presented significantly higher values than plant-free sites (Figures 3, 4; HSD test, *p* < 0.001). The bottom of the vegetated stands was generally undersaturated: often hypoxic (<50%) and frequently below <20%. Average O_2_ values measured at the surface of vegetated stands were <100% in spring and autumn, while in summer they were >100%. In this season, CO_2_ values measured at the surface of vegetated stands averaged 228 ± 39 ppmv and reached very low values (down to 8 ppmv). At vegetated stands, DIC mean values were of 0.8 ± 0.4, 0.6 ± 0.4, and 0.8 ± 0.3 mM (surface+bottom pooled data) for spring, summer, and autumn, respectively. At plant-free areas, DIC mean values were of 0.4 ± 0.3, 0.5 ± 0.1, and 0.4 ± 0.1 mM for spring, summer, and autumn, respectively.

CH_4_ values did not vary seasonally at vegetated neither at plant-free areas (Figures 3, 4 and Table 1); at vegetated stands, the water column was significantly stratified for CH_4,_ with mean values of 1.92 ± 0.91 μM (from 0.05 to 38.7 μM) and 13.71 ± 31.97 μM (from 0.05 to 227 μM), measured at the surface and at the bottom, respectively (HSD test, *p* < 0.001). At the bottom of vegetated stands, values were significantly higher values than at the bottom of plant-free sites (HSD test, *p* < 0.001), where CH_4_ averaged 0.20 ± 0.06 μM and 0.19 ± 0.05 μM, for the surface and the bottom, respectively. Overall, values at plant-free sites were comprised between 0.05 and 0.67 μM.

**FIGURE 5:**
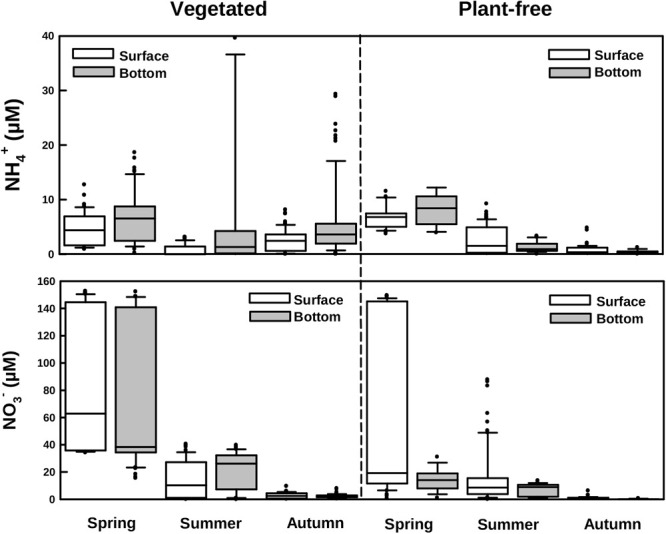
Boxplots of ammonium and nitrate measured along different seasons in vegetated and plant-free areas at the surface and the bottom of the water column.

Dissolved inorganic nitrogen varied seasonally, with NO_3_^-^ declining from spring to autumn, at both vegetated and plant-free areas. Values were significantly higher at vegetated sites; nevertheless, no stratification of the water column was detected (Figures 3, 5 and Table 1). At vegetated stands, NO_3_^-^ values averaged 78.1 ± 51.1, 19.5 ± 14.0, and 2.5 ± 1.7 μM (surface + bottom pooled data) for spring, summer, and autumn, respectively. At plant-free sites, NO_3_^-^ values averaged 20.0 ± 17.8, 16.4 ± 20.0, and 0.8 ± 1.2 μM (surface + bottom pooled data) for spring, summer, and autumn, respectively. NH_4_^+^ values varied seasonally at both vegetated and plant-free sites, with a marked stratification in the vegetated water column (Figures 3, 5 and Table 1). Here, surface values were comprised between 0.01 and 12.7 μM (mean 2.7 ± 0.6 μM), while bottom values were comprised between 0.01 and 86.9 μM (mean 7.0 ± 4.3 μM). At plant-free sites, values ranged from 0.1 to 11.5 μM (mean 3.4 ± 0.5 μM) and from 0.1 to 12.0 μM (mean 3.2 ± 0.4 μM), at the surface and at the bottom, respectively.

**FIGURE 6:**
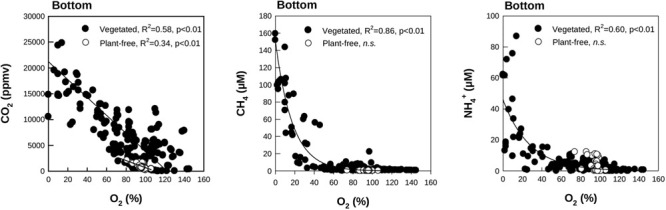
Scatter plots of O_2_ and CO_2_ (Left), CH_4_ (Middle), and NH_4_^+^ (Right) measured at the bottom of the vegetated and plant-free areas (seasonal data pooled). Regressions are calculated through a linear model for CO_2_, and through an exponential model for CH_4_ and NH_4_^+^. *n.s.* indicates a not significant relationship.

**FIGURE 7:**
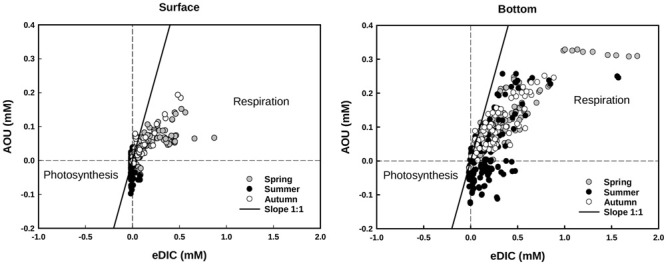
Apparent oxygen utilization (AOU) and excess of dissolved inorganic carbon (eDIC) plotted according different seasons at the surface and at the bottom of the vegetated stands. Respiration and remineralization processes are reflected in positive values of AOU and eDIC, whereas the effects of photosynthesis are reflected in negative values. The graphical representation takes inspiration on Dinauer and Mucci (2017).

In general, CO_2_, CH_4_, and NH_4_^+^ concentrations at the bottom layer of the water column were inversely dependent on O_2_ values for vegetated stands (Figure 6), whereas their relationship was never significant for values measured at the surface. In vegetated stands, AOU at the surface ranged from -0.10 (summer) to 0.20 mM (autumn), with mean values comprised between -0.01 ± 0.03 and 0.5 ± 0.5 mM, measured in summer and autumn, respectively. At the bottom, values were comprised between -0.13 (summer) and 0.33 mM (spring), with mean values ranging from 0.02 ± 0.09 to 0.11 ± 0.09 mM, measured in summer and spring, respectively. eDIC at the surface ranged from -0.01 (summer and spring) to 0.52 mM (autumn), with mean values comprised between 0.00 ± 0.02 and 0.17 ± 0.17 mM, measured in summer and spring, respectively. At the bottom, eDIC values ranged between -0.01 (summer and autumn) to 1.78 mM (spring), with mean values ranging from 0.19 ± 0.23 and 0.38 ± 0.35 mM, measured in summer and spring, respectively. The relationship between AOU and eDIC in vegetated stands (Figure 7), evidenced a prevalence of respiration processes at the bottom all year round, while photosynthesis was prevailing during summer at the surface of colonized areas.

For DOC, TN, and TP, values varied seasonally, with no significant differences between surface and bottom values (Tables 1, 2). At vegetated stands, DOC values were significantly lower than those measured at plant-free sites at all seasons except in autumn (HSD test, *p* < 0.001).

**Table 2:** Dissolved organic carbon (DOC), total nitrogen (TN), and total phosphorus (TP) measured along different seasons in vegetated stands and plant-free areas at the surface and at the bottom of the water column (mean ± SD).

		Vegetated		Plant-free	
		Surface	Bottom	Surface	Bottom
DOC (mg L^-1^)	Spring	12.3 ± 0.3	12.4 ± 0.5	14.2 ± 1.6	14.9 ± 0.9
	Summer	13.2 ± 0.4	13.1 ± 0.4	13.9 ± 0.9	14.4 ± 0.9
	Autumn	13.5 ± 0.5	13.6 ± 0.7	13.4 ± 0.8	13.8 ± 1.2
TN (mg L^-1^)	Spring	0.7 ± 0.2	0.8 ± 0.2	0.6 ± 0.2	–
	Summer	0.5 ± 0.1	0.7 ± 0.3	0.6 ± 0.1	–
	Autumn	1.1 ± 0.1	1.3 ± 0.5	1.2 ± 0.4	–
TP (mg L^-1^)	Spring	0.06 ± 0.07	0.06 ± 0.06	0.02 ± 0.01	–
	Summer	0.06 ± 0.04	0.07 ± 0.03	0.02 ± 0.01	–
	Autumn	0.08 ± 0.09	0.12 ± 0.06	0.06 ± 0.04	–

### Seasonal Nutrients and Carbon Budget

In vegetated stands, the height of the vegetation and the relative proportion of occupation in the water column varied according to the season (one-way ANOVA, *p* < 0.001, *F*_2,22_), with relatively short stems in winter and spring, when plants occupied about 44% of the water depth (Figure 3), which turned longer and thicker in summer and autumn (56 and 52% of the water depth). In some cases, the vegetated stands occupied 80% of the water column height; shoot density varied between 22 ± 17 to 407 ± 475 shoots m^-2^, measured in winter and summer, respectively.

Vegetated stands were mainly constituted by *Egeria densa* (at least 80% of the biomass at each station), whereas *Lagarosiphon major* occurred only in few stations with a much lower biomass. Biomass values, as well as GPP, DD, and NPP, varied seasonally at all stations, with a marked increase in summer and autumn compared to spring and winter (Table 3A). Biomass ranged from 46 to 1339 g_DW_ m^-2^, measured in winter and summer, respectively. When transforming GPP, DD, and NPP in nutrients uptake, loss, and fixation, respectively, we can estimate that dense vegetated stands fix a positive amount of nutrients, on an annual scale (Table 3B). When upscaling those values to the vegetated areas within the lake, on an annual basis, we can estimate that vegetated stands fix 2319 ± 1196 tons C year^-1^, 97 ± 50 tons N year^-1^ and 19 ± 10 tons P year^-1^ during their growth.

**Table 3:** **(A)** Total biomass (measured), gross primary production, decomposition, and net primary production (estimated) for vegetated stands of *E. densa* and *L. major* at different seasons. **(B)** Nutrients mobilization (uptake, calculated from GPP; loss, calculated from DD; fixation, calculated from NPP) within dense mats of *E. densa* and *L. major* at stand and lake scales.

(A)	Total biomass	GPP	DD	NPP
	g_DW_ m^-2^	g_DW_ m^-2^d^-1^	g_DW_ m^-2^d^-1^	g_DW_ m^-2^d^-1^
Spring	319 ± 245	11.7 ± 5.2	–5.2 ± 2.3	6.5 ± 2.9
Summer	668 ± 414	76.5 ± 38.7	–32.8 ± 16.6	36.4 ± 18.4
Autumn	567 ± 537	36.9 ± 26.2	–20.1 ± 14.3	16.8 ± 11.9
Winter	87 ± 50	1.6 ± 1.0	–1.2 ± 0.7	0.4 ± 0.3
				

**(B)**		**Carbon**	**Nitrogen**	**Phosphorus**
		**g C m^-2^year^-1^**	**g N m^-2^year^-1^**	**g P m^-2^year^-1^**

Stand scale	*Uptake*	4105 ± 2125	171 ± 89	34 ± 18
	*Loss*	–1920 ± 1007	–80 ± 42	–16 ± 8
	*Fixation*	1949 ± 1005	81 ± 42	16 ± 8

		**tons C year^-1^**	**tons N year^-1^**	**tons P year^-1^**

Lake scale	*Uptake*	4885 ± 2528	204 ± 105	41 ± 21
	*Loss*	–2285 ± 1198	–95 ± 50	–19 ± 10
	*Fixation*	2319 ± 1196	97 ± 50	19 ± 10

**FIGURE 8:**
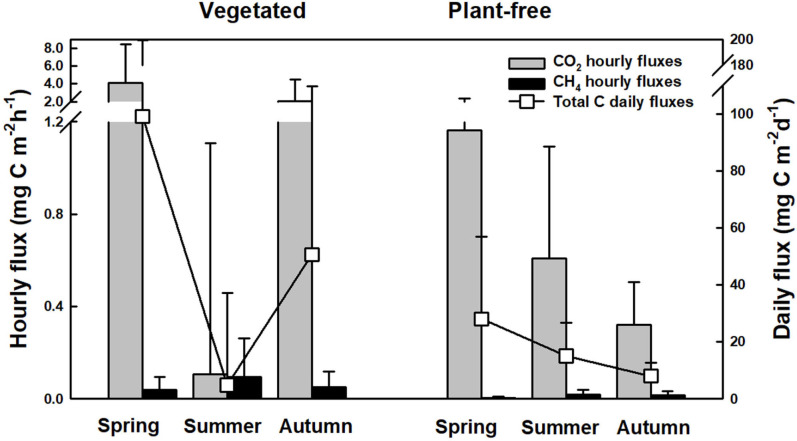
Hourly and total diffusive carbon fluxes (from CO_2_ and CH_4_) calculated from concentrations measured at the surface of the water column of vegetated and plant-free areas.

Coherently with concentrations measured at the surface of the water column, diffusive carbon fluxes calculated at the water–air interface followed a seasonal pattern (Figure 8). At vegetated stands, the highest value was recorded in spring (99.2 ± 104.8 mg C m^-2^d^-1^) and the lowest in summer (4.9 ± 32.3 mg C m^-2^d^-1^); at plant-free sites, the highest value was recorded in spring (28.0 ± 28.9 mg C m^-2^d^-1^) and the lowest in autumn (8.0 ± 4.6 mg C m^-2^d^-1^). Overall, the major contribution to diffusive carbon fluxes was given by CO_2_, and only in a minor part by CH_4_, with the summer period at vegetated sites as solely exception. At the annual scale, during the growing season of the plants (March to November), we can estimate that vegetated stands release 13.9 ± 1.2 g C m^-2^ year^-1^, while plantfree sites release 4.6 ± 0.3 g C m^-2^ year^-1^. When upscaling to the lake scale, we can estimate that dense vegetated stands emit 17 ± 1 tons C per growing season, whereas plant-free areas emit, in the same period, an estimated amount of 69 ± 4 tons C.

## Discussion

### Biogeochemical Functioning in Invasive Macrophyte Stands

The first evidence of our study is that the presence of a massive biomass development of invasive plants generates the stratification of the water column. The thermal and chemical stratification of the water column has been reported in a few studies concerning other submerged aquatic vegetation (Mazzeo et al., 2003; Andersen et al., 2017). Vilas et al. (2017) found that water stratification due to extensive colonization of *Potamogeton crispus* started when plants occupied more than 50% of the water column, which is a value that is overpassed in most of our samplings. Nevertheless, the variability measured within our dataset could be given by episodic wind events disturbance on sediment resuspension and water mixing. Conversely, water stagnation and related extreme values can be attributable to prolonged and extremely calm weather conditions (Søndergaard et al., 1992; Gale et al., 2006). Water stratification/mixing is also dependent on the biomass degree and stand ages of the sites, which can present a variable quantity of organic matter buildup in the sediment and thus influence the degree of respiration and consumption of oxygen (Boros et al., 2011).

The stratification of dissolved oxygen has important cascade effects on the local biogeochemistry, and in particular on the dynamics of carbon and nitrogen. In the dark bottom of the water column, under a thick layer of stems, plants respiration is not compensated by an equivalent oxygen release (Tavechio and Thomaz, 2003). The elevated heterotrophic respiration rates, filled up by the availability of labile dead biomass, generate here important amounts of DIC. This carbon is diffused to the water column surface, where it can be emitted toward the atmosphere as CO_2_. This process appears to be connected to the seasonal variations in net primary production and biomass decomposition of the plants. Indeed, unlike the plant-free areas, carbon emissions from vegetated stands are accentuated in spring and autumn, and much lower in summer. Coherently, the negative eDIC values measured in summer in the euphotic layer above the canopy indicate that this carbon is likely re-utilized by photosynthesis and only partially diffused toward the surface and then toward the atmosphere. In summer, the CO_2_ undersaturation measured at the surface indicates indeed a complete sequestration of CO_2_ in the water column. This is typical of freshwater systems where the biogeochemical functioning is seasonally determined by the ecophysiology of dense macrophyte mats (Bowes et al., 1979; Wang et al., 2006; Pierobon et al., 2010).

The quantity of DIC present in the whole water column is just satisfactory to support the daily primary production of the plants. Indeed, in summer, plants present a NPP of 36.4 ± 18.4 g_DW_ m^-2^d^-1^, which corresponds to a fixation of 13.1 ± 6.6 g C m^-2^d^-1^. If we consider the mean value of 0.6 ± 0.4 mM DIC measured in summer for the whole water column, for a fixed volume of water having a base of 1 m^2^ and an average height of 2.8 ± 0.4 m, we can estimate that the water column contains about 20 g C. This quantity is likely entirely consumed in a day by the plants in summer, as the carbon loop constituted by respiration-uptake is very fast and relates of an efficient coupling between bacterial nutrients regeneration and assimilation by plant shoots. Thus, the bottom part of the water column acts as a source of carbon, whereas the canopy of plants at the surface acts as a sink. In spring and autumn, the lower NPP do not allow the sequestration of the whole DIC generated from the organic matter degradation. This approximation is confirmed by the eDIC values, indicating the prevalence of respiration processes in the bottom of the vegetated water column all over the year, and in spring and autumn also at the surface.

The oxygen consumption and the settlement of hypoxic/anoxic conditions favor the production and buildup of methane and ammonium. Methanogenic bacteria develop thanks to the availability of dissolved organic matter (DOM) derived from the decomposition of decaying biomass (Zhang et al., 2018). A part of this methane is possibly consumed by aerobic methanotrophy within the canopy of the plants near the surface (Yoshida et al., 2014). However, a large amount of CH_4_ is diffused toward the surface of the water column, that contributing to carbon emissions from the vegetated stands. Nevertheless, calculated carbon diffusive fluxes are much lower than those measured in other systems colonized by floating-leaved invasive plants (Pierobon et al., 2010; Ribaudo et al., 2012; Oliveira-Junior et al., 2018). That confirms that, despite the elevated concentrations measured at the bottom of the water column, the CH_4_ oxidation and the CO_2_ uptake within submerged-leaved plants can significantly reduce the net effect on carbon emissions. Under hypoxic and anoxic conditions, the nitrification process is limited (Seitzinger, 1988), so that NH_4_^+^ accumulates in the lower layer of water column. As in the case of CO_2_, during summer, NH_4_^+^ can be efficiently taken up by dense vegetation. Indeed, a mean value of 6 ± 13 μM NH_4_^+^ measured for the whole water column, corresponds to a quantity of about 0.22 g N for a water column of 2.8 ± 0.4 m high. This quantity is completely depleted by primary production, as the corresponding NPP in summer is of 36.4 ± 18.4 g_DW_ m^-2^d^-1^, and thus is equivalent to a fixation of 0.5 ± 0.3 g N m^-2^d^-1^. Inversely, in spring and autumn, the dissolved nitrogen regenerated from decomposition cannot be wholly exhausted by fixation into biomass.

### Biogeochemical Processes From Local to Lake Scale

Invasive species are known to colonize new areas thanks to peculiar ecophysiological adaptations, such as fast growth rates and phenotypic plasticity, and to the availability of resources and space (Riis et al., 2010; Gillard et al., 2017). As pristine conditions, the lake investigated in this study is characterized by low concentrations of phosphorus and nitrogen (Cellamare et al., 2012): furthermore, the total nutrients discharge from the watershed has been reduced during the past decades thanks to the management of rural activities (Buquet et al., 2017). Nevertheless, the extended photoperiod and mild temperatures characterizing the region, coupled to the availability of space and the absence of other canopy-forming hydrophytes, constitute a very favorable unsaturated ecological niche for aquatic weeds to spread. The settlement of invasive hydrophytes in originally oligo-mesotrophic systems is reported elsewhere (Nagasaka, 2004; Mjelde et al., 2012; Bolpagni et al., 2015), that contrasting with the general tendency of the establishment of invasive plants in meso-eutrophic freshwaters (Kelly and Hawes, 2005; Hussner et al., 2009). Our study shows that the presence of such a massive area of primary production and biomass decomposition affects the carbon budget of the whole lake. Indeed, if we extend the carbon diffusive fluxes calculated for vegetated and plant-free areas to their correspondent surface area (1.19 and 15.01 km^2^, respectively), it results that, during their vegetative growth, plant stands release three fold more C per surface area than plant-free sites. Although they cover only 7% of the lake area, plant stands contribute to 20% of the lake carbon emissions and constitute a hotspot of carbon release (Wang et al., 2006). On the opposite, in Lacanau Lake, dense beds formed by aquatic weeds could be responsible for the storage of a part of the nutrients incoming the lake from the catchment area (Reddy et al., 1987; Søndergaard et al., 2003). During the year 2014, the total mass balance of the lake (including inputs from the watershed and the unvegetated sediments) results in a net storage of 67 tons N year^-1^ and 0.16 tons P year^-1^ within the lake (Buquet et al., 2017). We here estimate that invasive vegetated stands require more than 200 tons N year^-1^ and 40 tons P year^-1^ for their gross primary production, but this quantity is not available in the lake, neither coming from the watershed, neither originating from benthic fluxes in unvegetated areas (Buquet et al., 2017). Concomitantly, the fast renewal (NPP) and decay (DD) of biomass result in the almost total reutilization of the nutrients regenerated from the sediment, and in a small part of nutrients stocked in the sediment, through sediment accretion. In correspondence with vegetated stands, we observe indeed an accumulation of low-density, highly organic fluffy sediments (Bertrin et al., 2017). The predominance of recycled sedimentary N and P is well known in marine systems for seagrasses growth (Bartoli et al., 1996; Deborde et al., 2008). Carvalho et al. (2005) suggest that due to the rapid decomposition of *Egeria* spp. at high temperatures, a very small exportation of nutrients is expected from its stands to distant regions of the lake. Effectively, DOC measured in vegetated stands being lower than those of plant-free areas, we can expect that a process of priming effect boosts benthic bacterial communities, fueled by the continuous supply of fresh labile plant material, and rapidly degrading organic matter to inorganic compounds (Findlay et al., 1986; Marion and Paillisson, 2003). Bini et al. (2010) also commented that, in dense *Egeria* spp. vegetated stands, the nutrients regeneration is ephemeral, temporally limited to the vegetative growth of the plants, and spatially restricted to the areas of the lake that are colonized. Our results show that effectively, in summer, this could be the case. Nevertheless, the seasonal investigation performed on water chemistry shows an excess of nutrients during the seasons of slow plant growth. Especially in spring, when nutrients regeneration is elevated and not fully compensated by fixation into biomass, the nutrients export from vegetated to plant-free areas could be possible thanks to a low shoot density allowing the water circulation during strong wind events (Losee and Wetzel, 1993; James and Barko, 1994; Bertrin et al., 2017). In addition, according to the elevated concentrations of TN and TP measured in autumn, fresh organic matter deposition, reduced light and dynamic redox conditions in plant stands would make these areas potential temporary sources of available P for phytoplankton in plant-free areas (Buquet et al., 2017). Unfortunately, the sampling strategy adopted in this study was not designed for, from a spatial and a temporal point of view, detecting any diffusive gradient of nutrients between the vegetated stands and unvegetated areas. Moreover, investigations focusing on the sediments underlying the plants are needed to complete the current knowledge.

## Conclusion

In this study, we show that, once settled, *Egeria densa* and *Lagarosiphon major* are able to act as ecological engineers and modify the sites they colonize, by keeping a not-limiting nutrients level along the year, thanks to the formation of dense stands where a constant production of labile organic matter stimulates the microbial loop. We here show that in vegetated areas of the lake, the whole nutrients level is generally higher than in the rest of the lake, that seasonal fluctuations of oxygen and carbon are intensified and that an impact on the whole-lake ecosystem functioning is possible.

## Author Contributions

CR, VB, GA, PA, and JT-R conceived and designed the research project. CR, GJ, AJ, and DB collected the field data. All the co-authors commented on and approved the final manuscript.

## Conflict of Interest Statement

The authors declare that the research was conducted in the absence of any commercial or financial relationships that could be construed as a potential conflict of interest.

## Supplementary Material

APPENDIX S1 Dates and hours of sampling at different sites of the Lacanau Lake. The complete physicochemical database is reported.
