# Corrigendum: Invasive Aquatic Plants as Ecosystem Engineers in an Oligo-Mesotrophic Shallow Lake

**DOI:** 10.3389/fpls.2021.656314

**Published:** 2021-03-08

**Authors:** Cristina Ribaudo, Juliette Tison-Rosebery, Damien Buquet, Gwilherm Jan, Aurélien Jamoneau, Gwenaël Abril, Pierre Anschutz, Vincent Bertrin

**Affiliations:** ^1^EA 4592 Géoressources et Environnement, ENSEGID, Pessac, France; ^2^Irstea, UR EABX, Centre de Bordeaux, Cestas, France; ^3^CNRS UMR 5805 Environnements et Paléoenvironnements Océaniques et Continentaux, Université de Bordeaux, Pessac, France; ^4^Biologie des Organismes et Ecosystèmes Aquatiques, Muséum National d'Histoire Naturelle, Paris, France; ^5^Programa de Geoquímica, Universidade Federal Fluminense, Niterói, Brazil

**Keywords:** carbon emission, methane, hypoxia, water stratification, nutrients regeneration, seasonal, primary production, exotic plants

In the original article, there was a mistake in Figure 8 as published. An error was made while converting carbon flux values from moles to grams. As a consequence, the original figure showed diffusive carbon fluxes which were lower than real ones. The corrected Figure 8 appears below.

**Figure 8:**
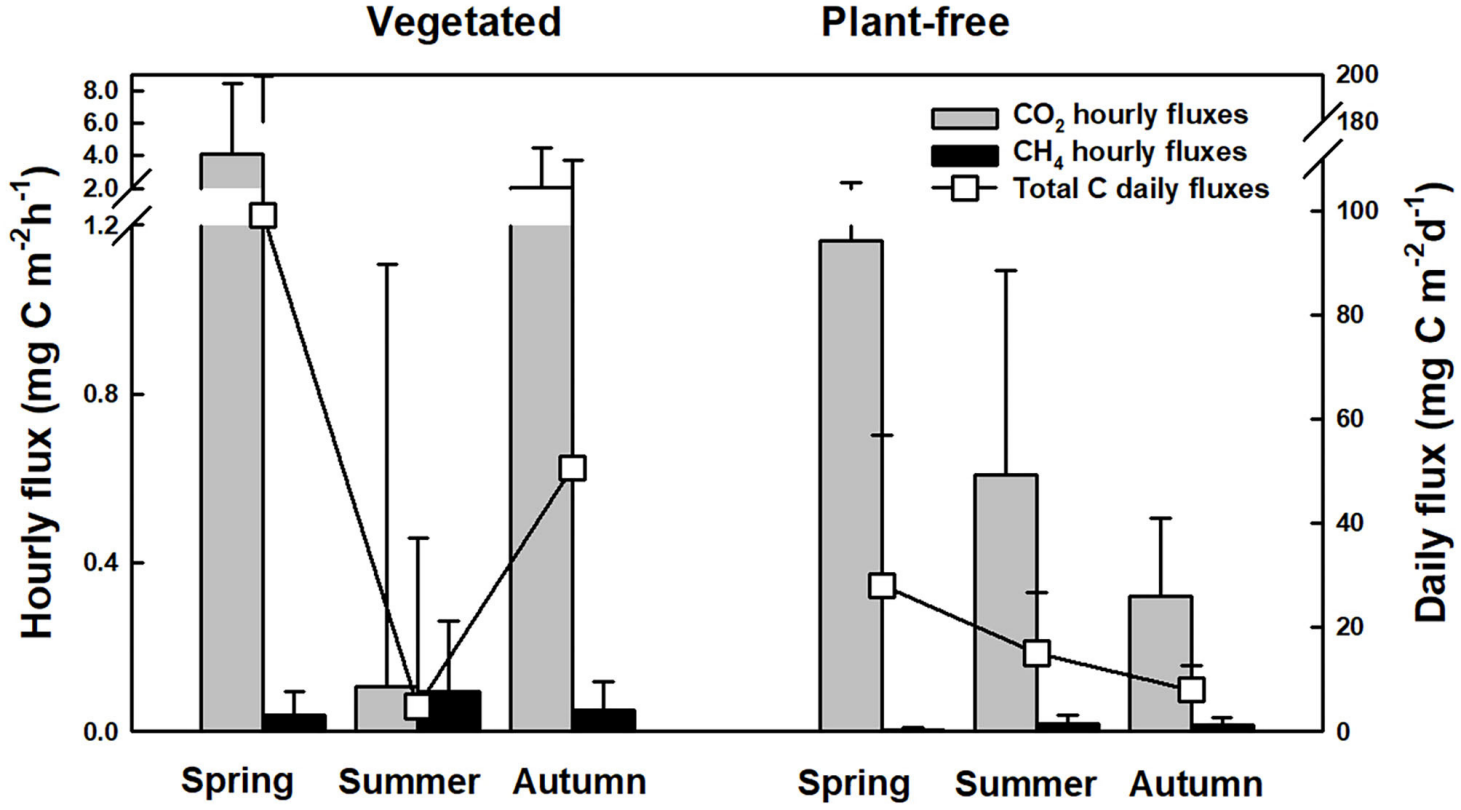
Hourly and total diffusive carbon fluxes (from CO_2_ and CH_4_) calculated from concentrations measured at the surface of the water column of vegetated and plant-free areas.

Further, due to the same error outlined above, the carbon budget extended to the annual period and lake scale was incorrect.

A correction has therefore been made to the **Results** section, subsection **Seasonal Nutrients and Carbon Budget**, paragraph three:

“Coherently with concentrations measured at the surface of the water column, diffusive carbon fluxes calculated at the water–air interface followed a seasonal pattern (Figure 8). At vegetated stands, the highest value was recorded in spring (99.2 ± 104.8 mg C m^−2^ d^−1^) and the lowest in summer (4.9 ± 32.3 mg C m^−2^ d^−1^); at plant-free sites, the highest value was recorded in spring (28.0 ± 28.9 mg C m^−2^ d^−1^) and the lowest in autumn (8.0 ± 4.6 mg C m^−2^ d^−1^). Overall, the major contribution to diffusive carbon fluxes was given by CO_2_, and only in a minor part by CH_4_, with the summer period at vegetated sites as solely exception. At the annual scale, during the growing season of the plants (March to November), we can estimate that vegetated stands release 13.9 ± 1.2 g C m^−2^ year^−1^, while plant-free sites release 4.6 ± 0.3 g C m^−2^ year^−1^. When upscaling to the lake scale, we can estimate that dense vegetated stands emit 17 ± 1 tons C per growing season, whereas plant-free areas emit, in the same period, an estimated amount of 69 ± 4 tons C.”

The authors apologize for this error and state that this does not change the scientific conclusions of the article in any way. The original article has been updated.

